# Are Higher-Order Constructs in Evolutionary Psychology Attributable to Omitted Cross-Loading Bias? An Exploratory Structural Equation Modeling Approach

**DOI:** 10.1007/s12110-025-09497-7

**Published:** 2025-07-22

**Authors:** George B. Richardson, Daniel G. Bates, Laura E. McLaughlin, Nathan McGee, Winnie W.-Y. Tse, Mark H. C. Lai

**Affiliations:** 1 School of Human Services, University of Cincinnati, Cincinnati, OH USA; 2 Department of Psychology, University of Southern California, Los Angeles, CA USA

**Keywords:** Big Five, General factor of personality, Trait emotional intelligence, K-factor, Exploratory structural equation modeling, Omitted cross-loading bias

## Abstract

Global constructs such as the general factor of personality (GFP), trait emotional intelligence (TEI), and the K-factor have generated considerable interest as well as controversy in evolutionary psychology. Research employing exploratory structural equation modeling (ESEM) suggests higher-order factors may be attributable to the omission of cross-loadings from confirmatory factor models and scale score computation, which can upwardly bias first-order factor and scale score correlations. In the current project, we conducted two studies to determine if GFP and TEI are method artifacts using national random-digit-dialing (*n* = 1,805) and teacher (*n* = 331) samples, respectively. We also conducted a study examining the possibility that K is an artifact using a sample of college students (*n* = 661). Using ESEM and bifactor ESEM to allow cross-loadings, we found evidence suggesting a general factor did not subsume all the Big Five personality traits and concluded that GFP is likely an artifact of omitted cross-loading bias. Evidence of global K and TEI factors survived free estimation of cross-loadings, and findings suggest total TEI scores may be sufficient; however, model-based reliability was too low to warrant the use of total Mini-K scores. Researchers should consider using ESEM to examine the internal structures of their scales at the item level before computing total scale scores.

**Supplementary Information:**

The online version contains supplementary material available at 10.1007/s12110-025-09497-7.

Many studies in evolutionary psychology have examined the higher-order or “general” factors. For instance, numerous studies and systematic reviews have extracted a general factor of personality (GFP) from responses to measures of the Big Five personality traits (e.g., van der Linden et al., 2018; Wu et al., 2021). The GFP has become the subject of extensive evolutionary theorizing (e.g., see Chua et al., 2021; Dunkel et al., 2018, 2020; Figueredo et al., 2004; van der Linden et al., 2018; Wu et al., 2020) as well as considerable debate, with some researchers arguing that it is a substantively meaningful factor that indexes social effectiveness (e.g., see Dunkel & van der Linden, 2014) or life history strategy (Figueredo & Rushton, 2009), and others suggesting it is a method artifact resulting from the omission of facet-level correlations and/or socially desirable responding (e.g., see Muncer, 2011). Proponents of evolutionary perspectives on GFP have also extracted a general factor from trait emotional intelligence (TEI) subscales (Figueredo et al., 2012; van der Linden et al., 2017). Like GFP, TEI has been theorized to be an indicator of slow life history strategy (van der Linden et al., 2015).

The K-factor is yet another global construct that has received attention in evolutionary psychology. Studies of K, which was theorized to index individual differences in human life history speed, have proliferated during the past few decades (for a review, see Black et al., 2017). Initially measured using scales administered in the Midlife in the United States (MIDUS) study and later using the popular Mini-K short form (Figueredo et al., 2006), the K-factor has subsumed indicators such as planning and control, social contact and support, community integration, attachment, religiosity, and altruism in studies using both exploratory factor analysis (EFA; e.g., Figueredo et al., 2005) and confirmatory factor analysis (CFA; e.g., Richardson et al., 2017). Psychometric critiques of K have raised many of the criticisms previously leveled at GFP (Copping et al., 2014, 2017; Richardson et al., 2017, 2021) and theoretical (Gruijters & Fleuren, 2018) and evolutionary genetic (Zietsch & Sidari, 2020) critiques of the conceptualization of K as life history speed have also emerged.

Richardson et al. (2021) argued that K does not measure life history speed yet deserves more theoretical and empirical attention given its replicability in terms of structure and plausibility as a proximate (rather than ultimate) cause of its indicators. Rather than indexing an over-arching pattern of investment in fitness components, K might measure social-cognitive variation stemming from the default mode network (DMN) and manifesting as investments in interpersonal relationships with intimate partners and other conspecifics, planning, empathic responding, and so on (Richardson et al., 2021). Consistent with their proximate cause theorizing about K, the authors found evidence that the structure of K was measurement invariant by sex in a national sample of adults. Subsequently, they laid out several promising avenues for researchers wishing to scrutinize the validity of K further.

While substantial evidence appears consistent with the existence of the global factors identified above, it remains possible that GFP, TEI, and K are all method artifacts due to *omitted cross-loading bias*. Omitted cross-loadings can create the appearance of general factors when none are present by inflating facet correlations (as discussed further below; Marsh et al., 2014). This omitted cross-loading bias has not yet received attention in evolutionarily-informed discussions of GFP (van der Linden et al., 2021), TEI (van der Linden et al., 2016), or K (Black et al., 2017); perhaps because exploratory structural equation modeling, which allows both cross-loadings and model testing, is not yet widely known in evolutionary psychology or the broader field of evolution and human behavior.

## Exploratory Structural Equation Modeling

Scale score and CFA summaries of responses to multi-item questionnaires are ubiquitous in social and behavioral science, yet research rarely acknowledges their strict and often unrealistic assumptions (Lasker et al., 2022; Marsh et al., 2014; van der Sluis et al., 2010). Key among these is unidimensionality, the assumption that responses to each item reflect one major factor (van der Sluis et al., 2010). Methodologists have expressed skepticism regarding this assumption, or the expectation that a simple structure can be achieved, since Thurstone’s pioneering work on exploratory factor analysis (Morin et al., 2020). Recognition that it might be too strict eventually led to the development of exploratory structural equation modeling (ESEM; Asparouhov & Muthén, 2009; Marsh et al., 2014; Morin et al., 2013), which enables researchers to relax the unidimensionality assumption by allowing each item to load on all factors (Marsh et al., 2014). ESEM represented a major advance beyond traditional EFA because like structural equation modeling, it enabled researchers to estimate standard errors, residuals, and residual covariances; evaluate model fit information; test measurement invariance (e.g., across groups or time); and embed measurement models within full structural models (Asparouhov & Muthen, 2009; Marsh et al., 2009). It also represented a major advance beyond CFA-based approaches to allowing cross-loadings (e.g., see Ashton et al., 2009) because of its greater robustness to the presence of cross-loadings that are unknown and/or large in number. Researchers (e.g., Ashton et al., 2009) previously identified cross-loadings via modification indices when they were not specified by prior research or theory, an approach that tends to overfit models to samples. Importantly, ESEM is also less subject to identification problems than CFA-based approaches.

ESEM solutions may be compared to CFA models and analyses of scores to determine the impact of omitting cross-loadings. The comparison between ESEM and CFA, in particular, is meaningful because (a) simulation evidence suggests ESEM recovers unbiased estimates of parameters such as factor correlations when cross-loadings are present as well as when they are absent (i.e., when unidimensionality actually does hold; Asparouhov et al., 2015); and (b) although ESEMs are less restrictive than CFAs, and could be expected to exhibit superior fit for that reason, several fit indices include a penalty for complexity that enables comparisons of more and less parsimonious models (e.g., the Root Mean Error of Approximation; Marsh et al., 2005; Marsh et al., 2009; Morin et al., 2020). Researchers can also use ESEM to embed EFA models within full structural models to further compare of EFA- and CFA-based measurement models in the context of broader nomological networks (Cronbach & Meehl, 1955).

Emerging ESEM research has provided clear evidence that unidimensionality frequently fails to hold and that ignoring even small cross-loadings (e.g., β = .10) can bias both within- and between-instrument correlations, whether scores or latent variables in CFA models are used (Asparouhov et al., 2015; Mai et al., 2018; Marsh et al., 2010). Key to the current study, analyses of Big Five personality inventories revealed that allowing cross-loadings yields better model fits (e.g., TLI = .731 vs. .893 and RMSEA = .044 vs. .028, respectively) and substantially smaller facet correlations (e.g., *r* = −.502 vs. −.205, respectively, for the correlation between neuroticism and extraversion; Booth & Hughes, 2014; Chiorri et al., 2016; Marsh et al., 2010). These findings are consistent with the results of a study that used a CFA-based approach (as previously discussed) and found that allowing cross-loadings decreased correlations among Big Five factors (Ashton et al., 2009). Also key to the current study, Perera (2015) examined a measure of emotional intelligence and found that, as with measures of Big Five personality traits, an ESEM solution fit much better than a CFA model (e.g., CFI = .936 versus .843) and the factor correlations were inflated in the CFA. Self-control, for instance, was correlated with sociability at *r* = .667 in the CFA but at only *r* = .116 in the ESEM solution. Similar findings have emerged in studies of constructs such as attention-deficit-hyperactivity disorder (ADHD; Gomez & Stavropoulos, 2021), psychopathy (Cooke & Sellbom, 2019), resilience (Askeland et al., 2020), post-traumatic stress disorder (Petri et al., 2022), depression, anxiety, stress (Gomez et al., 2020), and eating disorder (Costello et al., 2023).

The evidence reviewed above suggests efforts to carefully develop, evaluate, and refine multi-item scales (for a primer on scale development, see Boateng et al., 2018) have not been sufficient to address the difficulty inherent in writing items that reflect only one entity (McGrath, 2005; Morin et al., 2016). In social and behavioral science, this is partly due to the standard practice of first using EFA to identify the internal structure of questionnaire items and then using CFA to test the fit of the identified structure against data from independent samples. Moving from EFA to CFA, researchers typically adopt the strict unidimensionality assumption (i.e., item responses reflect only one underlying factor) and retain items as indicators of factors only if their standardized EFA loadings exceeded conventional rules of thumb (e.g., $\beta \ge .30$ or $\beta \ge .40$; Brown, 2015). Items without any loadings above the chosen threshold may be discarded. If an item has a loading on a (primary) factor that exceeds the threshold as well as loadings on additional (secondary) factors that fall below it (i.e., *cross-loadings*), the former are typically estimated in CFA, and the latter, omitted. In practice, the use of factor-loading cutoffs tends to eliminate small cross-loadings.

Omitted cross-loadings are significant because they can compromise inference in several ways. They can cause poor fit, leading researchers to erroneously conclude that the number of dimensions suggested by EFA is incorrect. As noted previously, they can also inflate first-order factor correlations (Marsh et al., 2014). This can occur in CFA even when the exclusion of cross-loadings does not produce poor model fits according to conventional rules of thumb (Marsh et al., 2014). When this bias inflates facet correlations and, in turn, the variance that can be attributed to general factors, researchers may use fewer scores than is necessary to summarize item covariance (Morin et al., 2016).

## Current Project

Evidence suggests the GFP (e.g., Chiorri et al., 2016), TEI (Perera, 2015), and K (Richardson et al., 2017) may be method artifacts due to omitted cross-loading bias. To our knowledge, only one study has directly tested this hypothesis with respect to GFP (Ashton et al., 2009) and no studies have tested it with respect to TEI or K. Although some studies of the K-factor did use ESEM, they examined scale scores (Richardson et al. (2021) or used the previously discussed rule of thumb ($\beta \ge .30)$ to discard loadings in the move from ESEM to CFA (Richardson et al., 2017). Consequently, they were not able to address the possibility of omitted item-level cross-loadings. It is worth noting, however, that Richardson et al. (2017) did report ESEM first-order factor correlations in their first study and CFA factor correlations in their second study. Although these sets of correlations were not directly comparable, being estimated from independent samples, the pattern of results was the same as observed in ESEM studies of GFP and TEI. The fit of the ESEM solution was slightly better (e.g., CFI = 1.00) than the fit of the CFA (e.g., CFI = .97) and the correlations in the ESEM were smaller. For instance, insight, planning, and control were correlated with mother/father relationship quality at *r* = .259 in the CFA but at only *r* = .078 in the ESEM solution.

The current project uses ESEM to address the gaps identified above, with the objective of determining whether three global constructs of significance in evolutionary psychology—GFP, TEI, and K—are method artifacts attributable to omitted cross-loading bias. In this effort, we extend upon previous ESEM research by using bifactor ESEM (Morin et al., 2016) to directly test whether higher-order general factors are consistent with the data. We conduct three studies to achieve our objective. In the first, we examine the structure of Big Five personality traits using data from a large national random digit dialing (RDD) sample of adults from the study of Midlife Development in the United States (MIDUS; *n* = 1,791). In the second, we examine the structure of TEI using data from teachers who participated in the National Center for Research on Early Childhood Education Teacher Professional Development Study (*n* = 331). In the third, we re-examine the structure of the Mini-K reported by Richardson et al. (2017) using their samples of college students (total *n* = 661).

## Study 1

### Methods

#### Data

Study 1 analyzed publicly available national data from the MIDUS study, which investigated the role of behavioral, psychological, and social factors in accounting for age-related variations in health and well-being.^1^ The MIDUS study is longitudinal with two rounds (1995–1996 and 2004–2006) and includes a (main) random digit dialing (RDD) sample, twin and sibling samples, and city oversamples. The current study is limited to RDD sample participants (*n*_Round 1_ = 3,487) who completed the Round 2 self-administered questionnaire (SAQ) containing the Big Five personality scales (*n* = 1,805). Participant ages ranged from 30 to 84 years ($\mu$ = 56.85) and 54.7% identified as female. The sample was 89.4% White, 4.9% Black and/or African American, 1.7% Native American or Aleutian Islander/Eskimo, .6% Asian, 0.1% Native Hawaiian or Pacific Islander, and 2.8% Other. The remaining 0.5% selected ‘don’t know’ or refused. Finally, 91.6% graduated high school, 7.8% graduated from a 2-year college or vocational school with an associate degree, 18.9% graduated college with a bachelor’s or master’s degree, and 4.7% earned a doctoral degree.

#### Instruments

##### The Midlife Development Inventory (MIDI) Personality Scales

The survey developers created the MIDI scales by selecting adjectives from existing trait lists and inventories (Bem, 1981; Goldberg, 1992; John, 1990; Trapnell & Wiggins, 1990) and then evaluated them using a probability sample of *n* = 1,000 men and women aged 30 to 70. Respondents indicated how well each personality item described them on a scale from 1 (*a lot*) to 4 (*not at all*). For the MIDUS 2 SAQ sample, Cronbach’s (1951) $\alpha$ was acceptable across the scales (neuroticism [$\alpha$ = 0.74], extroversion [$\alpha$ = 0.76], openness [$\alpha$ = 0.77], conscientiousness [$\alpha$ = 0.69], and agreeableness [$\alpha$ = 0.81]). The MIDI scales have been used in hundreds of studies of outcomes ranging from depression (Goodwin & Gotlib, 2004) to sleep (Sutin et al., 2020) and inflammation (Luchetti et al., 2014).

#### Analysis

We conducted ESEM and CFA analyses (Asparouhov & Muthén, 2009) using the MPlus 8 software package and the robust weighted least squares (WLSMV) estimator because items were ordinal (Muthén et al., 1997). We used the delta parameterization and conducted all significance tests at the conventional $\alpha$ = 0.05 level. We used Geomin and Bi-Geomin rotations for our ESEM and bi-factor ESEM analyses, respectively. Because a recent study suggests Bi-Geomin rotation may recover bifactor structures less well than approaches based on target rotation (Giordano & Waller, 2020), we examined the sensitivity of our results to the choice of rotation method by comparing Geomin and target rotated solutions. For a description of ESEM parameterization, see the Mplus User Guide Version 8, example 4.1 (http://www.statmodel.com/download/usersguide/MpluUserGuideVer_8.pdf). For more information about the bi-factor specification, specifically, see Morin et al. (2016).

##### Omitted Cross-Loading Bias

We compared estimates derived from both Geomin and target rotated ESEM solutions to estimates derived from CFA and scale scores. If non-trivial cross-loadings (β = .10; Asparouhov et al., 2015) were detected in the ESEMs and substantively important differences between the sets of results were observed, we interpreted this as evidence of practically significant bias due to the strict unidimensionality assumption imposed in CFA and analysis of scores (i.e., in the CFA or independent cluster models, items were only allowed to load on their target factor). We then tested bifactor models to more directly examine whether general factors were consistent with the data. In this analysis, we evaluated the plausibility of general factors via model fit, patterns of loadings on general factors, model-based reliability for general factors as indexed by Omega hierarchical (Ω > 0.80 suggests scores capture adequate variance in general factors; Reise et al., 2013), and the degree to which specification of the general factor reduced the number of cross-loadings. We note that Omega hierarchical is not meaningful for specific factors because all items load on them and many small loadings are theoretically expected, such that $\Omega$ will be quite small by design.

##### Model Fit

We used a variety of fit indices that provide different information about model fit. We considered the substantive meaningfulness of the model, non-significance of the χ^2^ likelihood ratio statistic (Bollen, 1989), comparative fit index (CFI) values greater than or equal to 0.95 (Hu & Bentler, 1999), and root means square error of approximation (RMSEA) values of less than or equal to 0.05 (Browne & Cudeck, 1992) as evidence of acceptable fit to the data. We focused on CFI and RMSEA in our comparisons of ESEMs to CFA models because they include penalties for model complexity (one and the ratio of χ^2^ to degrees of freedom, respectively).

### Results

#### ESEM

Based on prior research, we specified and tested a five-factor ESEM model using Geomin rotation and fit to the data was inadequate (χ^2^ = 1686.57[205], *p* < .001; CFI = .948; RMSEA = 0.064[0.061-0.066]). The fit of a six-factor model was adequate (χ^2^ = 1099.78[184], *p* < .001; CFI = 0.968; RMSEA = 0.053[0.050-0.056]); however, only two items (B1SE6N and B1SE6Q) loaded on the sixth factor. Thus, we returned to the five-factor model, observed modification indices, and found a relatively large index (> 200), suggesting a residual covariance between the two items should be specified. We added this specification, and the fit of the resulting five-factor model was slightly better than that observed for the six-factor model (χ^2^ = 1082.34[204], *p* < .001; CFI = 0.969; RMSEA = 0.049[0.046-0.052]). Accepting the five-factor solution, we interpreted its loadings (see Table 1). All items had substantial ($\beta \ge .30$) loadings on their expected factors, and loadings ranged from $\beta$ = 0.34 to 0.87. Across the items, there were 35 cross-loadings larger than $\beta$ = 0.10 and 14 greater than $\beta$ = 0.20.

**Table 1:**
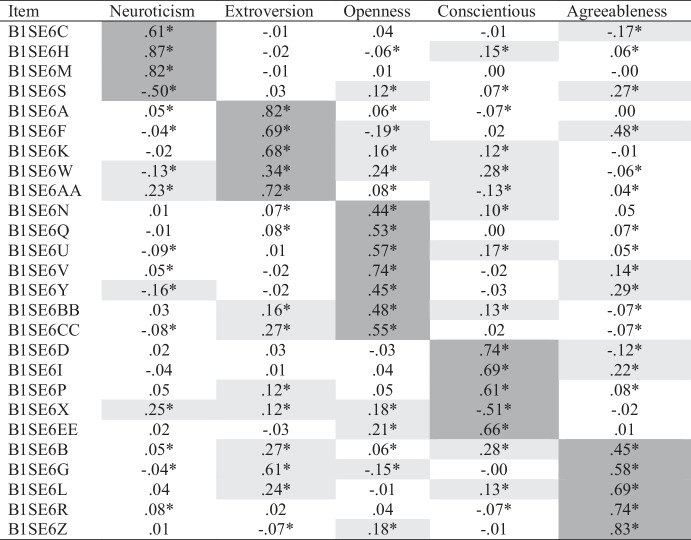
Big Five ESEM Loadings

Table displays standardized loadings. *p $\le$ .05. Expected loadings are highlighted in dark gray. Cross-loadings $\ge$ .10 are highlighted in light gray

We compared the Geomin rotated solution to that produced by a target rotation and found no substantively important differences in structure (see *Supplementary Materials*). We then used CFA to test the five-factor model and fit was poor (χ^2^ = 4727.70[288], *p* < .001; CFI = 0.845; RMSEA = 0.093[0.090-0.095]). The magnitudes of the loadings were consistent with past research (see *Supplementary Materials*).

Next, we compared the Geomin and target-rotated ESEM, CFA, and scale score correlations (see Table 2). The CFA produced larger estimates in every case, and in two instances, the CFA correlations were at least twice as large as their Geomin and target-rotated ESEM counterparts. For half of the correlations, the Geomin and Target rotations produced estimates substantively smaller than CFA and scale scores. For the other half, the two ESEM solutions produced correlations with magnitudes similar to those observed for the scale scores. Perhaps the most striking discrepancy between approaches was the correlation between neuroticism and agreeableness, which was near zero in both ESEM solutions and nonsignificant under the Geomin rotation. The pattern of small correlations with neuroticism that emerged from all but the CFA approach suggested a GFP was implausible, but it appeared particularly implausible in the ESEMs.

**Table 2:**
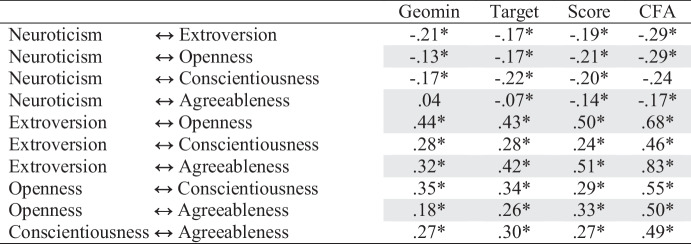
Comparison of Big Five ESEM, subscale score, and CFA correlations

Gray highlighting denotes correlations larger for both scores and CFA than for the ESEM solutions

#### Bifactor ESEM

Next, we specified a bifactor ESEM to examine the plausibility of a GFP directly. This model included a bifactor (GFP) and five specific factors (Neuroticism, Extroversion, Openness, Conscientiousness, and Agreeableness). We tested this model using the Bi-Geomin rotation and fit to the data appeared superior to the five-factor ESEM (CFI = 0.981; RMSEA = 0.041[0.038-0.044]). However, the χ^2^ test was still significant (χ^2^ = 729.73[183], *p* < .001) and specification of the GFP did not decrease the number of cross-loading; instead, it resulted in the addition of one. Observing the specific factor loadings (Table 3), we saw that items intended to measure neuroticism, extroversion (except B1SE6W), conscientiousness, and agreeableness all loaded substantially on their specific factors ($\beta$ s > 0.30). The openness loadings, however, ranged from $\beta$ = 0.03 to 0.29 and three of seven were non-significant, indicating that once the GFP was accounted for, little trait-specific variance remained.

**Table 3:**
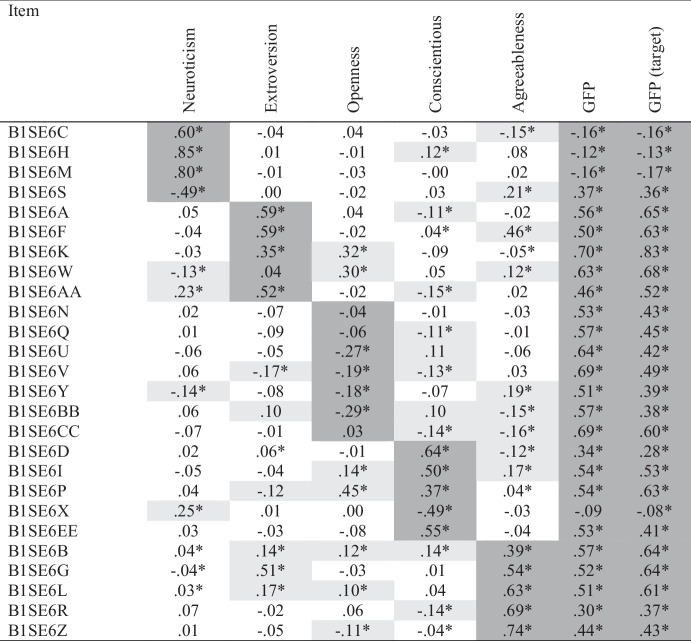
Big Five Bifactor ESEM Loadings

Table displays standardized loadings. *p $\le$ .05. Primary loadings are highlighted in dark gray. Cross-loadings $\ge$.10 are highlighted in light gray

All items except one loaded significantly on the GFP, and all but four loadings were larger than $\beta$ = 0.30. Only one neuroticism item had a loading on the GFP that was moderate in size. Three of the four neuroticism items had loadings on GFP that were smaller than $\beta$ = 0.20. The GFP explained six percent or less of the variances in those three items. We evaluated the reliability of general factor scores and found that omega hierarchical was almost adequate (Ω = 0.789), suggesting GFP score variance largely reflects differences on the general factor. As noted, however, the factor itself did not adequately subsume neuroticism, implying that GFP scores also did not adequately summarize individual differences in that trait.

We compared the Geomin and target-rotated solutions and found that whereas a trait-specific openness factor did not emerge under the Bi-Geomin rotation, this factor did emerge as expected under the target rotation. No other substantive differences in the pattern of loadings were observed. Target-rotated GFP loadings are displayed in the rightmost column of Table 3, and all the other parameters are presented in *Supplementary Materials*.

### Discussion

Using ESEM and Geomin rotation, we found the expected five-factor personality trait structure. We also found 35 non-trivial cross-loadings that could bias trait covariances. Consonantly, most scale score correlations were larger than their ESEM counterparts, and all CFA correlations were larger than their ESEM counterparts (sometimes twice as large). Three of four ESEM correlations between neuroticism and other traits were trivial in size, and the correlation with agreeableness was non-significant. Consistent with the pattern of ESEM correlations, our bifactor ESEM revealed that neuroticism did not adequately reflect the general factor. Importantly, our ESEM and bifactor ESEM findings were consistent across Geomin and target rotations, with the one exception being the presence of a trait-specific openness factor under target but not Bi-Geomin rotation.

Ultimately, our findings replicate past research (Ashton et al., 2009) and suggest that the GFP is an artifact of omitted cross-loading bias or the inflation of trait covariances when cross-loadings are ignored. In particular, the CFA and scale score correlations between agreeableness and neuroticism appeared wholly due to bias. Researchers interested in GFP should examine their data using ESEM before computing scores. If our findings replicate, prior evidence about GFP (e.g., van der Linden et al., 2021) requires re-examination and evolutionary theorizing about its variation (Dunkel et al., 2018; Figueredo et al., 2004; van der Linden et al., 2018; Wu et al., 2020) may be moot.

## Study 2

### Methods

#### Data

The Teacher Professional Development Study was a randomized evaluation of coursework and consultation support delivered to teachers.^2^ Study participants worked in state and local preschool programs across ten locations in eight states. Teachers were selected to participate in one of four possible conditions: A) Course/Consultancy; B) Course/No Consultancy; C) No Course/Consultancy; and D) No Course/No Consultancy. The course was delivered in Phase 1 of the study, and in Phase 2, the consultancy was delivered. Overall, 427 teachers participated in Phase 1, and 401 teachers participated in Phase 2. Pertinent to the current study, the researchers administered the Self-Rated Emotional Intelligence Scale (SREIS; Brackett et al., 2006) as a part of the Phase 2 pretest.

Study 2 analyzed the data from 331 teachers who participated in Phase 1. Four hundred twenty-seven teachers participated in Phase I, and 401 teachers participated in Phase 2. As a result of missing data and attrition and subsequent supplemental recruitment of participants for phase 2, a sample of 411 participants involved in the treatment/control groups was administered the SREIS and personality measures. Participants in our analytic sample were aged 19–69 years ($$\mu$$ = 42.23), and 93.4% were female. Income ranged from less than $15,000 to $150,000 + annually. Most participants were low (34.4%) and middle socioeconomic status (38.2%). The racial/ethnic composition of the sample was 49.1% Black/AA, 32.1% White/Caucasian, 6.3% Other Hispanic, 5.1% Puerto Rican, 4.1% Mexican American/Chicano, 3.4% Native American Indian, 1.5% Other Asian, 1% Filipino, 1% Chinese, 0.5% Asian Indian, 0.5% Other, 0.2% Japanese, 0.2% Korean, 0.2% Pacific Islander, and 0.2% Vietnamese.^3^ The Teacher Professional.

#### Instruments

##### Self-Rated Emotional Intelligence Scale (SREIS)

The SREIS is a 19-item scale that assesses perceived emotional abilities, with subscales specific to perception of emotions, use of emotions to facilitate thought, understanding of emotions, and management of emotions (Brackett et al., 2006). Items are rated on a response scale from 1 (*very inaccurate*) to 5 (*very accurate*). Example items include, “By looking at people’s facial expressions, I recognize the emotions they are experiencing,” and “I am the type of person to whom others go when they need help with a difficult situation” (Brackett et al., 2006, p. 795). Cronbach’s $\alpha$ for the entire scale was 0.77 in the original evaluation (Brackett et al., 2006).

#### Analyses

The Study 2 analytic approach was the same as in Study 1.

### Results

#### ESEM

Based on prior research (Bracket et al., 2006), we specified and tested a four-factor ESEM model. Fit to the data was adequate according to CFI but not the χ^2^ test or RMSEA (χ^2^ = 223.98[101], *p* < .001; CFI = 0.957; RMSEA = 0.061[0.050-0.071]). The fit of a five-factor model was substantially better (χ^2^ = 150.05[86], *p* < .001; CFI = 0.978; RMSEA = 0.047[0.035-0.060]); however, only two items (Emotion 15 and Emotion 16) loaded substantially (i.e., $\beta \ge$ 0.30) on the fifth factor and they were designed to measure different constructs (managing and perceiving emotions, respectively). Thus, this factor did not make substantive sense, and we returned to the four-factor model and interpreted its loadings (see Table 4). In contrast to the original study, we found that the items designed to measure managing emotions reflected two distinct factors (social and self, respectively). Moreover, whereas the understanding and perceiving emotions items reflected separate factors in the original study, they reflected a single factor in the current study. Finally, two of the using emotions items reflected their expected factor, but a third did not load significantly upon it. Across the items, there were 28 cross-loadings larger than $\beta$ = 0.10 and 12 greater than $\beta$ = 0.20. Compared to our previous Big Five ESEM analysis, we observed larger cross-loadings for the SREIS, with seven exceeding $\beta$ = 0.30 and three exceeding $\beta$ = 0.50.

**Table 4:**
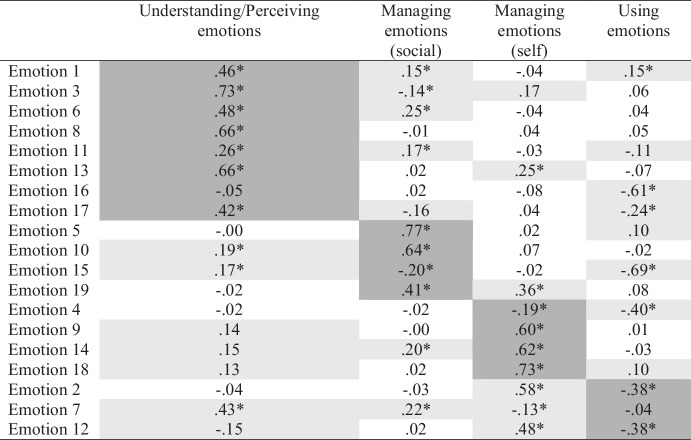
Emotional Intelligence ESEM Loadings

Table displays standardized loadings. *p $\le$ .05. Primary loadings are highlighted in dark gray. Cross-loadings $\ge$ .10 are highlighted in light gray

We did not use target rotation in this study given the hypothesized model failed to fit adequately. We did specify a CFA based on the Geomin-rotated ESEM solution and the conventional $\beta$> 0.30 cutoff to examine the impact of omitted cross-loadings on the factor correlations. The fit of this model was marginal (χ^2^ = 272.84[126], *p* < .001; CFI = 0.948; RMSEA = 0.059[0.050-0.069]). Factor loadings are displayed in *Supplementary Materials*.

Next, we observed the factor correlations for the four-factor ESEM solution and compared them to the correlations among the CFA factors and scale scores (see Table 5). Most scale score correlations (67%) exceeded their ESEM counterparts, if only slightly. However, the correlation between understanding/perceiving emotions and using emotions did not differ between approaches, and the correlation between managing emotions (social) and managing emotions (self) was smaller than its ESEM counterparts. In contrast to the Big Five findings, no striking differences between the correlations from the two approaches emerged despite the presence of many cross-loadings in the ESEM, some of which were large.

**Table 5:**
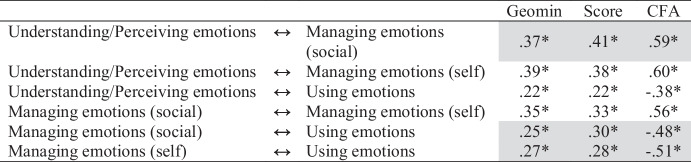
Comparison of TEI ESEM, subscale score, and CFA correlations

Gray highlighting denotes correlations larger for both scores and CFA than for the ESEM solution

#### Bifactor ESEM

We specified a bifactor ESEM to examine the plausibility of a general TEI factor more directly. This model included a bifactor (TEI) and four specific factors (Understanding/Perceiving emotions, Managing emotions [social], Managing emotions[self], and Using emotions). We tested this model and fit to the data appeared superior to the four-factor ESEM and identical to the five-factor ESEM (χ^2^ = 150.050[86], *p* < .001; CFI = 0.978; RMSEA = 0.047[0.035-0.060]). Moreover, specification of the general TEI factor reduced the number of cross-loadings to 18 (i.e., by 36%). Observing the factor loadings (Table 6), we saw that all but one item had a significant loading on the general factor, and only six loadings were not at least moderate in size (i.e., $\beta \ge$ 0.30). Turning to the specific factors, we found that only three of seven items that previously loaded significantly on understanding/perceiving emotion still did so, and all but one loading was small. Only one managing emotions (social) item still loaded significantly on its factor, whereas all the managing emotions (self) items still had moderate to large and significant factor loadings. Similar findings were observed for using emotions. Thus, the TEI covariance seemed due to a general factor and two specific factors (managing emotions-self and using emotions). We evaluated the reliability of general factor scores and found that omega hierarchical was almost adequate (Ω = 0.749), suggesting TEI score variance largely reflects differences on the general factor, meaning the SREIS can likely be considered essentially unidimensional and total scores should be useful.

**Table 6:**
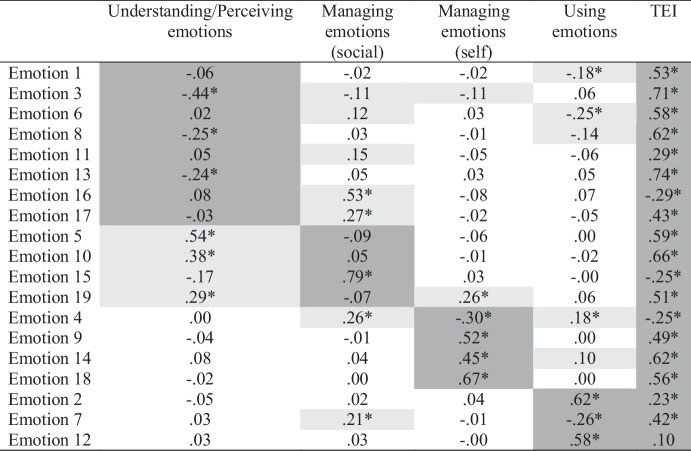
Trait Emotional Intelligence Bifactor ESEM Loadings

Table displays standardized loadings. *p $\le$ .05. Expected loadings are highlighted in dark gray. Cross-loadings $\ge$ .10 are highlighted in light gray

### Discussion

Using ESEM, we found a different four-factor TEI structure than Bracket et al. (2006). We also found 28 cross-loadings larger than $\beta$ = 0.10, which could bias trait covariances. Although most scale score correlations were larger than their ESEM counterparts, the differences in size were trivial. In contrast to the findings for GFP, our bifactor ESEM revealed that TEI items were largely subsumed by a general factor, with two of the specific factors no longer subsuming most of their items once the general TEI variance was controlled. Taken together, these findings suggest that TEI may not be an artifact of omitted cross-loading bias or the inflation of trait covariances when cross-loadings are ignored, and a single factor or score may be sufficient.

## Study 3

### Methods

#### Data

Richardson et al. (2017) conducted two studies of data from independent samples of college students from a large Midwestern university. Participants were recruited through their classes, both online and face-to-face, as well as through a subject pool. Participants did not receive any form of incentive for their participation. Students read an information form, provided consent, and subsequently completed a questionnaire. Overall, the participants sampled were relatively diverse with respect to socioeconomic status. More than 20% of the students at the university were first-generation college students, suggesting the institution served an economically diverse student body. Moreover, 58 (8.8%) had primary childhood residence total household incomes below US$25,000 (the 2013 poverty line for a two-child family was ~ US$24,000, Federal Interagency Forum on Child and Family Statistics, 2015), 63 (9.5%) reported that their families were worse off financially than most, 244 (36.9%) reported that their father had a high school level education or less, and 199 (30.1%) indicated the same for mother’s educational attainment (the U.S. percentage for both mothers and fathers in 2013 was 30%, Kena et al., 2015). Please see Richardson et al. (2017) for more detailed descriptions of the Study 1 and 2 samples. The current study used the pooled sample to re-examine the structure of the Mini-K (*n* = 661).

#### Instruments

##### Mini-K

As described in Richardson et al. (2017), the Mini-K is a 20-item brief scale designed to measure the domains captured by the Arizona Life History Battery (ALHB; Figueredo et al., 2006). These domains include (a) insight, planning, and control; (b) mother/father relationship quality; (c) friends social contact and support; (d) family social contact and support; (e) harm avoidance; and (f) community involvement. Two to three items reflect each of these constructs. The Mini-K has demonstrated adequate internal consistency in past research (Olderbak et al., 2014).

#### Analyses

In study 3, we used the same ESEM procedures as in Studies 1 and 2.

### Results

#### ESEM

Based on prior research (Richardson et al., 2017), we specified and tested a six-factor ESEM model and fit to the data was excellent (χ^2^ = 66.32[39], *p* = .004; CFI = 0.996; RMSEA = 0.033[0.018-0.046]). All items had significant and moderate to large loadings on their expected factors (Table 7), except one (Item 5, $\beta$ = 0.29). Across the items, there were 15 cross-loadings larger than $\beta$ = 0.10 and two greater than $\beta$ = 0.20. Compared to our previous ESEM analyses, we observed fewer and smaller cross-loadings, with only one exceeding $\beta$ = 0.30. Notably, one-third of cross-loadings were loadings on community involvement.

**Table 7:**
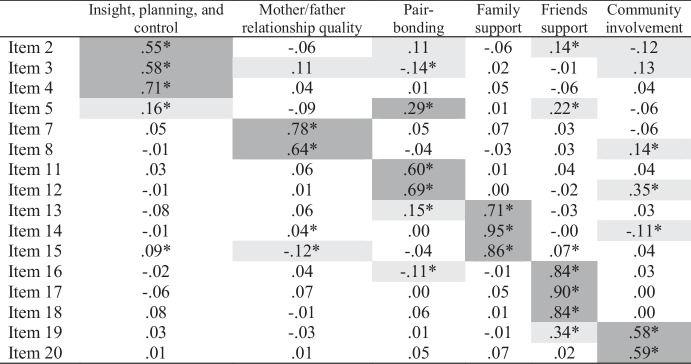
Mini-K ESEM Loadings (Geomin Rotation)

Table displays standardized loadings. *p $\le$ .05. Expected loadings are highlighted in dark gray. Cross-loadings $\ge$ .10 are highlighted in light gray

We compared the Geomin rotated solution to that produced by a target rotation and found no substantively important differences in structure (see *Supplementary Materials*). We then used CFA to test the five-factor model, and fit was adequate according to CFI but not the χ^2^ test or RMSEA (χ^2^ = 253.69[89], *p* < .001; CFI = 0.976; RMSEA = 0.053[0.045-0.061]). The magnitudes of the loadings were consistent with past research (see *Supplementary Materials*).

Next, we compared the Geomin and target rotated ESEM, CFA, and scale score correlations (see Table 8). The CFA produced larger estimates in every case. However, the scale scores produced estimates similar in magnitude to, and in some cases smaller than, the Geomin and target rotated solutions. This is not necessarily surprising given correlations among scores are often attenuated due to unreliability. Overall, the Geomin rotation tended to produce the smallest correlations, the CFA produced the largest, and the target rotation and scale score correlations fell in between. In the Geomin rotated solution, three non-significant correlations suggested a general K-factor was implausible. However, a general factor appeared more plausible based on the correlations produced by the target rotation.

**Table 8:**
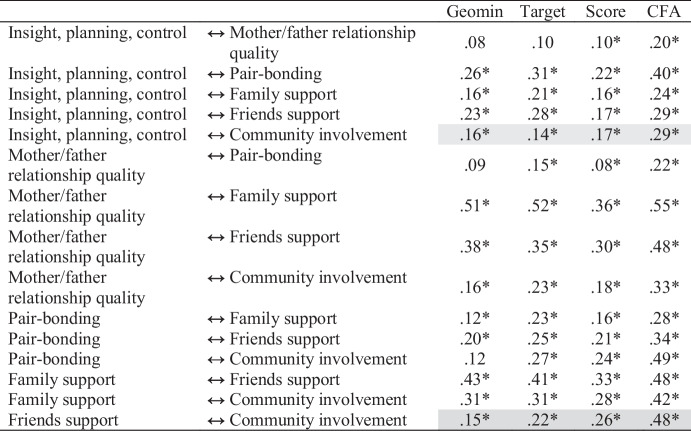
Comparison of Mini-K ESEM, subscale score, and CFA correlations

Gray highlighting denotes correlations larger for both scores and CFA than for the ESEM solutions

#### Bifactor ESEM

We specified a bifactor ESEM to examine the plausibility of a general K factor more directly. This model included a bifactor (K) and six specific factors (see Table 9). We tested this model using Geomin rotation and fit to the data appeared slightly better than for the six-factor ESEM (χ^2^ = 33.75[29], *p* = .25; CFI = 0.999; RMSEA = 0.016[0.000-0.035]). Moreover, specification of the K-factor reduced the number of cross-loading to 10 (i.e., by 33%). This model was also comparable to, and fit the data better than, the higher-order CFA that Richardson et al. (2017) reported in their previous study (see Study 3 results; χ^2^ = 270.390[112], *p* < .001; CFI = 0.977; RMSEA = 0.046[0.039-0.053]). For instance, ΔCFI = 0.022 and the χ^2^ test was non-significant for the bifactor ESEM but significant for the higher-order CFA. Observing the factor loadings (Table 9), we saw that nine of 20 had moderate (i.e., $\beta \ge$ 0.30) to large loadings on the general factor, and seven loadings were non-significant. However, the non-significant loadings were all *non*-trivial in size ($\beta$ s = 0.19 to 0.46) and statistically significant under target rotation (as discussed below).

**Table 9:**
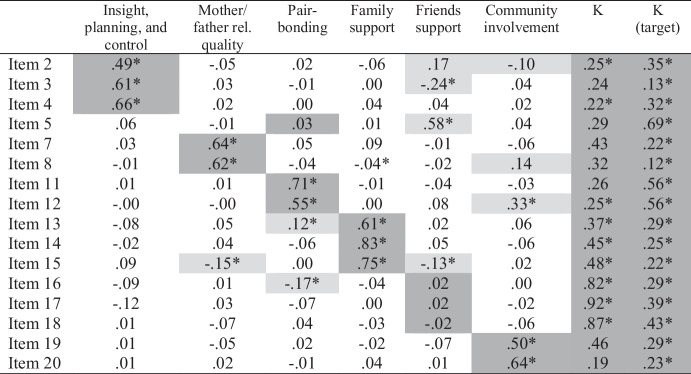
Mini-K Bifactor ESEM Loadings (Bi-Geomin Rotation)

Table displays standardized loadings. *p $\le$ .05. Expected loadings are highlighted in dark gray. Cross-loadings $\ge$ .10 are highlighted in light gray. General factor loadings from target rotated solution in K (target) column

Consistent with the first-order Geomin-rotated ESEM, the social support item loadings on K were all moderate to large (although the mother/father relationship quality were among those not statistically significant), and the friend support item loadings were largest ($\beta$ s = 0.82 to 0.92). All friend support item loadings on their specific factor were near zero and non-significant. The K-factor loadings for the insight, planning, and control items; pair-bonding items; and one of the community involvement items were small. These findings suggested the general factor might be identified as friend support. However, we estimated the bifactor ESEM again using target rotation and found more similar loadings on the general factor across the items and all were significant (see the rightmost column in Table 9; for all loadings, see *Supplementary Materials*); that is, the K-factor appeared more plausible once we encoded some theory into the estimation process. However, eight of 16 loadings (56%) were small (though at least four neared $\beta$ = 0.30). Moreover, when we evaluated the reliability of general factor scores, we found that Omega Hierarchical was low (Ω = 0.527), suggesting the Mini-K cannot be considered essentially unidimensional. That is, although a general factor is somewhat consistent with the data, it is not a sufficiently dominant source of the covariance among the items to allow researchers to ignore the specific factors and use only total scale scores.

### Discussion

Overall, our ESEM findings seem consistent with the presence of a K-factor but call into question the usefulness of total Mini-K scores. We found 15 cross-loadings larger than $\beta$ = 0.10, which could bias trait covariances, and the bias seemed to decrease correlations among social support factors while inflating correlations among the non-social support factors. Two correlations with mother-father relationship quality and one correlation with community involvement were trivial in size and non-significant in the Geomin-rotated ESEM solution. We found similar results under target rotation. Consistent with the pattern of first-order ESEM factor correlations, our Geomin-rotated bifactor ESEM revealed that non-social support items tended to have small loadings on the global K-factor while friend support items had very large loadings upon it. Findings more consistent with a global factor emerged from the target-rotated solution, suggesting the K-factor examined in prior research may not be an artifact of omitted cross-loading bias, or the inflation of trait covariances when cross-loadings are ignored. However, model-based appears too low to warrant the use of total Mini-K scores.

## General Discussion

Global constructs such as the GFP, TEI, and the K-factor have generated considerable interest as well as controversy in evolutionary psychology. Research (e.g., Morin et al., 2016) employing ESEM suggests global factors can be attributable to omission of cross-loadings from CFA models and scale score computation, which can upwardly bias first-order factor and scale score correlations. In the current project, we used ESEM to determine if GFP and TEI are method artifacts using national random digit dialing and teacher samples, respectively. We also examined the possibility that K is an artifact using a sample of college students. Using ESEM and bifactor ESEM to allow cross-loadings, we did not find clear evidence of GFP and conclude it may be due to omitted cross-loading bias as suggested by prior research (Ashton et al., 2009). Evidence of global TEI and K factors, however, seemed to survive free estimation of cross-loadings. Whereas model-based reliability suggested total TEI scores may be sufficient, reliability for K was too low to warrant the use of total Mini-K scores.

Our findings provide some important implications for research attendant to higher-order or “global” constructs. The first is that researchers should avoid global factors including GFP until additional studies examine the internal structures of scales at the item level using ESEM. While it is increasingly clear that omitted cross-loading bias impacts CFA studies of personality, it is not yet apparent how many social and behavioral constructs are affected. Writing social and behavioral items that reflect only one construct is difficult, implying that cross-loadings are to be expected (Marsh et al., 2014).

Much social and behavioral research has examined individual subscales via factor analysis. Based on single-factor solutions, these studies have either proceeded to analyze subscale scores or included subscale items as reflective indicators of single factors in broader SEMs. These approaches assume a congeneric model (i.e., items load *only* on their factor) and can result in the omission of cross-loadings (Lasker et al., 2022). When scores are analyzed, omitted cross-loadings can bias trait covariances, as shown in the studies reported here, although unreliability and opposing omitted cross-loadings may counteract this bias to some degree. CFA might, in fact, be more subject to this kind of bias given opposing cross-loadings cannot be canceled out via aggregation and the estimator will “try” to minimize the fit function by absorbing them into the between factor covariances (Lasker et al., 2022; Rhemtulla et al., 2020). Because omitted cross-loading bias may affect correlations among subscale scores in multiple ways (as shown in the current study), we cannot identify a priori whether global factors will emerge due to this bias or tend to be obscured. More research using ESEM is needed to determine what big picture emerges if any.

Our findings suggest omitted cross-loading bias joins the likes of *p*-hacking and HARKing (Kerr, 1998; Open Science Collaboration, 2015; Smaldino & McElreath, 2016); lack of formal theorizing (Muthukrishna & Henrich, 2019; Smaldino, 2020); overrepresentation of samples from undergraduate and WEIRD populations (Pollet & Saxton, 2019; see also Gurven et al., 2013; Laajaj et al., 2019); and lack of measurement invariance testing (Lasker et al., 2022; Wang et al., 2018) in threatening the validity of inferences in evolution and human behavior. While careful attention to complex measurement models might seem onerous at times, any such investment of resources can produce significant savings by reducing opportunity costs. If GFP is a method artifact, what is the cost of all the hours spent studying it and theorizing about how it might be maintained in the population via selection? Time lost to studies of GFP could be allocated elsewhere.

### Limitations

Inference from the studies reported here is limited by our use of less representative samples in Study 2 and Study 3. It is not clear if the structure of TEI is invariant between teachers and the rest of the population. Moreover, the SREIS is not the most popular measure of TEI. Future research should examine if our findings extend to measures such as the Trait Emotional Intelligence Questionnaire (Petrides, 2001). Although the Mini-K has most often been administered to college samples, and the Richardson et al. (2017) samples were relatively diverse, it is not yet clear if similar findings would emerge in community- or population-based samples. More research is needed to replicate our results in more representative samples. Finally, we did not conduct invariance testing by sex, race/ethnicity, country of origin, and so on in the current project. Thus, it is unclear to what degree smaller cross-loadings might vary over groups and time.

## Supplementary Information

Below is the link to the electronic supplementary material.

Supplementary file1 (DOCX 44 KB)

## Data Availability

The data used in Study 1 and in Study 2 are freely available through the Interuniversity Consortium for Political and Social Science Research (ICPSR). https://www.icpsr.umich.edu/web/pages/. The data used in Study 3 are available from the authors upon request.

## Footnotes

1 For more information about the MIDUS samples or to access the data, please visit http://www.midus.wisc.edu/midus1/index.php.

2 For more information about the Teacher Professional Development Study or to access the data, please visit: https://www.icpsr.umich.edu/web/ICPSR/studies/34848#

3 Demographic percentages do not sum to 100% because participants could endorse multiple racial and ethnic categories.
