# Supplementary material for: Are Higher-Order Constructs in Evolutionary Psychology Attributable to Omitted Cross-Loading Bias? An Exploratory Structural Equation Modeling Approach
Source: Hum Nat. 2025 Jul 22;36(2):257–80. doi: 10.1007/s12110-025-09497-7 (PMC12417301; doi:10.1007/s12110-025-09497-7)
Supplement: Supplementary file 1 — Supplementary file1 (DOCX 44 KB) [file 12110_2025_9497_MOESM1_ESM.docx]

| Table 1  *Big Five ESEM Loadings (target rotation)* | | | | | |
| --- | --- | --- | --- | --- | --- |
| Item | Neuroticism | Extroversion | Openness | Conscientious | Agreeableness |
| B1SE6C | 0.611* | 0.015 | 0.048* | -0.036 | -0.106* |
| B1SE6H | 0.881* | -0.007 | -0.057* | 0.132* | 0.159* |
| B1SE6M | 0.823* | 0.004 | 0.009 | -0.014 | 0.088* |
| B1SE6S | -0.500* | -0.005 | 0.116* | 0.092* | 0.206* |
| B1SE6A | -0.029 | 0.775* | 0.106* | -0.107* | 0.032 |
| B1SE6F | -0.107* | 0.580* | -0.119* | -0.008 | 0.532* |
| B1SE6K | -0.078* | 0.646* | 0.183* | 0.097* | 0.003 |
| B1SE6W | -0.140* | 0.359* | 0.204* | 0.299* | -0.095* |
| B1SE6AA | 0.159* | 0.706* | 0.089* | -0.142* | 0.069* |
| B1SE6N | 0.008 | -0.152* | 0.870* | -0.091* | 0.076* |
| B1SE6Q | -0.006 | -0.148* | 0.970* | -0.183* | 0.087* |
| B1SE6U | -0.061 | 0.126* | 0.430* | 0.240* | -0.054* |
| B1SE6V | 0.043 | 0.116* | 0.546* | 0.083* | 0.045 |
| B1SE6Y | -0.140* | 0.035 | 0.353* | 0.039 | 0.208* |
| B1SE6BB | 0.032 | 0.260* | 0.367* | 0.176* | -0.134* |
| B1SE6CC | -0.087* | 0.342* | 0.457* | 0.067* | -0.147* |
| B1SE6D | 0.050* | -0.003 | -0.022 | 0.727* | -0.091 |
| B1SE6I | -0.014 | -0.022 | 0.010 | 0.725* | 0.221* |
| B1SE6P | 0.056* | 0.065* | 0.079* | 0.592* | 0.110* |
| B1SE6X | 0.223* | 0.177* | 0.145* | -0.500* | -0.027 |
| B1SE6EE | 0.053* | -0.020 | 0.161* | 0.684* | -0.006 |
| B1SE6B | 0.029 | 0.184* | 0.093* | 0.277* | 0.471* |
| B1SE6G | -0.109* | 0.492* | -0.075* | -0.028 | 0.629* |
| B1SE6L | 0.013 | 0.131* | 0.025 | 0.146* | 0.727* |
| B1SE6R | 0.067* | -0.087* | 0.076* | -0.062* | 0.772* |
| B1SE6Z | 0.013 | -0.119* | 0.147* | 0.047* | 0.808* |
| *Note*: Table displays standardized loadings. **p* $\leq$ .05. Expected loadings are highlighted in dark gray. Cross-loadings $\geq$ .10 are highlighted in light gray. | | | | | |

| Table 2  *Big Five CFA Loadings* | | | | | |
| --- | --- | --- | --- | --- | --- |
| Item | Neuroticism | Extroversion | Openness | Conscientious | Agreeableness |
| B1SE6C | 0.613* |  |  |  |  |
| B1SE6H | 0.752* |  |  |  |  |
| B1SE6M | 0.800* |  |  |  |  |
| B1SE6S | -0.736* |  |  |  |  |
| B1SE6A |  | 0.712* |  |  |  |
| B1SE6F |  | 0.921* |  |  |  |
| B1SE6K |  | 0.778* |  |  |  |
| B1SE6W |  | 0.625* |  |  |  |
| B1SE6AA |  | 0.585* |  |  |  |
| B1SE6N |  |  | 0.539* |  |  |
| B1SE6Q |  |  | 0.574* |  |  |
| B1SE6U |  |  | 0.669* |  |  |
| B1SE6V |  |  | 0.679* |  |  |
| B1SE6Y |  |  | 0.577* |  |  |
| B1SE6BB |  |  | 0.591* |  |  |
| B1SE6CC |  |  | 0.705* |  |  |
| B1SE6D |  |  |  | 0.574* |  |
| B1SE6I |  |  |  | 0.861* |  |
| B1SE6P |  |  |  | 0.737* |  |
| B1SE6X |  |  |  | -0.312* |  |
| B1SE6EE |  |  |  | 0.761* |  |
| B1SE6B |  |  |  |  | 0.760* |
| B1SE6G |  |  |  |  | 0.950* |
| B1SE6L |  |  |  |  | 0.833* |
| B1SE6R |  |  |  |  | 0.578* |
| B1SE6Z |  |  |  |  | 0.703* |
| *Note*: Table displays standardized loadings. **p* $\leq$ .05. | | | | | |

| Table 3  *Big Five Bifactor ESEM Loadings (target rotation)* | | | | | | |
| --- | --- | --- | --- | --- | --- | --- |
| Item | Neuroticism | Extroversion | Openness | Conscientious | Agreeableness | GFP |
| B1SE6C | .595* | -0.022 | 0.001 | -0.047* | -0.096* | -0.162* |
| B1SE6H | .848* | 0.034 | -0.022 | 0.104* | 0.145* | -0.125* |
| B1SE6M | .792* | 0.027 | 0.019 | -0.019 | 0.080* | -0.169* |
| B1SE6S | -.480* | -0.028 | 0.086* | 0.060* | 0.171* | 0.357* |
| B1SE6A | -.003 | 0.499* | 0.040 | -0.106* | -0.096* | 0.647* |
| B1SE6F | -.080* | 0.485* | -0.095* | 0.043 | 0.362* | 0.630* |
| B1SE6K | -0.036* | 0.180 | -0.031 | -0.060* | -0.142* | 0.830* |
| B1SE6W | -0.108* | -0.083 | 0.049* | 0.095* | -0.165* | 0.677* |
| B1SE6AA | 0.174* | 0.465* | 0.073* | -0.153* | -0.024 | 0.517* |
| B1SE6N | 0.025 | -0.059* | 0.313* | 0.029 | -0.001 | 0.426* |
| B1SE6Q | 0.008 | -0.077* | 0.373* | -0.070* | 0.017 | 0.454* |
| B1SE6U | -0.060* | 0.050 | 0.518* | 0.156* | -0.003 | 0.423* |
| B1SE6V | 0.060* | -0.101* | 0.570* | -0.077* | 0.092* | 0.491* |
| B1SE6Y | -0.140* | -0.036 | 0.369* | -0.039 | 0.218* | 0.389* |
| B1SE6BB | 0.038 | 0.203* | 0.482* | 0.127* | -0.104* | 0.377* |
| B1SE6CC | -0.066* | -0.029 | 0.363* | -0.094* | -0.153* | 0.601* |
| B1SE6D | 0.059* | 0.085* | 0.034 | 0.663* | -0.122* | 0.281* |
| B1SE6I | 0.003 | -0.097* | 0.001 | 0.548* | 0.145* | 0.530* |
| B1SE6P | 0.107* | -0.276* | -0.154* | 0.413* | -0.013 | 0.632* |
| B1SE6X | 0.216* | 0.004 | 0.089* | -0.502* | -0.007 | -0.077* |
| B1SE6EE | 0.065* | 0.015 | 0.208* | 0.591* | -0.022 | 0.406* |
| B1SE6B | 0.050* | 0.044 | -0.003 | 0.171* | 0.345* | 0.635* |
| B1SE6G | -0.080* | 0.412* | -0.070* | 0.017 | 0.451* | 0.637* |
| B1SE6L | 0.029 | 0.056* | -0.061* | 0.063* | 0.572* | 0.606* |
| B1SE6R | 0.074* | -0.105* | -0.026 | -0.127* | 0.670* | 0.372* |
| B1SE6Z | 0.014 | -0.073* | 0.144* | -0.019 | 0.738* | 0.427* |
| *Note*: Table displays standardized loadings. **p* $\leq$ .05. Primary loadings are highlighted in dark gray. Cross-loadings $\geq$.10 are highlighted in light gray. | | | | | | |

| Table 4  *Emotional Intelligence CFA Loadings* | | | | |
| --- | --- | --- | --- | --- |
|  | Understanding/Perceiving emotions | Managing emotions (social) | Managing emotions (self) | Using emotions |
| EMOTION1 | 0.550*  0.735*  0.614* |  |  |  |
| EMOTION3 |  |  |  |  |
| EMOTION6 |  |  |  |  |
| EMOTION7 | 0.421* |  |  |  |
| EMOTION8 | 0.665* |  |  |  |
| EMOTION13 | 0.801* |  |  |  |
| EMOTION17 | 0.421* |  |  |  |
| EMOTION5 |  | 0.735* |  |  |
| EMOTION10 |  | 0.821* |  |  |
| EMOTION19 |  | 0.357* |  |  |
| EMOTION2 |  |  | 0.617* |  |
| EMOTION9 |  |  | 0.701* |  |
| EMOTION12 |  |  | 0.473* |  |
| EMOTION14 |  |  | 0.818* |  |
| EMOTION18 |  |  | 0.821* |  |
| EMOTION19 |  |  | 0.358* |  |
| EMOTION2 |  |  |  | 0.539* |
| EMOTION4 |  |  |  | 0.563* |
| EMOTION15 |  |  |  | 0.598* |
| EMOTION16 |  |  |  | 0.603* |
| EMOTION12 |  |  |  | 0.516* |
| *Note*: Table displays standardized loadings. **p* $\leq$ .05. | | | | |

| Table 5  *Mini-K ESEM Loadings (Target Rotation)* | | | | | | |
| --- | --- | --- | --- | --- | --- | --- |
|  | Insight, planning, and control | Mother/father relationship quality | Pair-bonding | Family support | Friends support | Community involvement |
| Item 2 | 0.547* | -0.074 | 0.123* | -0.060 | 0.124* | -0.135* |
| Item 3 | 0.592* | 0.101* | -0.120* | 0.014 | -0.050 | 0.143* |
| Item 4 | 0.721* | 0.031 | 0.030 | 0.042 | -0.104* | 0.025 |
| Item 5 | 0.145* | -0.096 | 0.309* | 0.012 | 0.202* | -0.110 |
| Item 7 | 0.052 | 0.787* | 0.048 | 0.057 | 0.060 | -0.112* |
| Item 8 | 0.002 | 0.652* | -0.032 | -0.043 | 0.035 | 0.111* |
| Item 11 | 0.002 | 0.058 | 0.637* | 0.007 | 0.010 | -0.083 |
| Item 12 | -0.024 | 0.018 | 0.753* | 0.007 | -0.091* | 0.207* |
| Item 13 | -0.088* | 0.068 | 0.128* | 0.705* | -0.039 | 0.019 |
| Item 14 | -0.013 | 0.046 | -0.039 | 0.943* | 0.006 | -0.086* |
| Item 15 | 0.086* | -0.109* | -0.064* | 0.858* | 0.050 | 0.084* |
| Item 16 | -0.017 | 0.051 | -0.093* | 0.006 | 0.821* | 0.074* |
| Item 17 | -0.063* | 0.077* | 0.025 | 0.057* | 0.882* | 0.031 |
| Item 18 | 0.074* | -0.008 | 0.087* | 0.025 | 0.810* | 0.017 |
| Item 19 | 0.044 | -0.013 | 0.063 | 0.003 | 0.244* | 0.579* |
| Item 20 | 0.029 | 0.021 | 0.086* | 0.073 | -0.071 | 0.579* |
| *Note*: Table displays standardized loadings. **p* $\leq$ .05. Expected loadings are highlighted in dark gray. Cross-loadings $\geq$ .10 are highlighted in light gray. | | | | | | |

| Table 6  *Mini-K CFA Loadings* | | | | | | |
| --- | --- | --- | --- | --- | --- | --- |
|  | Insight, planning, and control | Mother/father relationship quality | Pair-bonding | Family support | Friends support | Community involvement |
| Item 2 | 0.533* |  |  |  |  |  |
| Item 3 | 0.614* |  |  |  |  |  |
| Item 4 | 0.691* |  |  |  |  |  |
| Item 5 |  | 0.851* |  |  |  |  |
| Item 7 |  | 0.633* |  |  |  |  |
| Item 8 |  |  | 0.429* |  |  |  |
| Item 11 |  |  | 0.589* |  |  |  |
| Item 12 |  |  | 0.734* |  |  |  |
| Item 13 |  |  |  | 0.744* |  |  |
| Item 14 |  |  |  | 0.909* |  |  |
| Item 15 |  |  |  | 0.859* |  |  |
| Item 16 |  |  |  |  | 0.817* |  |
| Item 17 |  |  |  |  | 0.944* |  |
| Item 18 |  |  |  |  | 0.857* |  |
| Item 19 |  |  |  |  |  | 0.796* |
| Item 20 |  |  |  |  |  | 0.520* |
| *Note*: Table displays standardized loadings. **p* $\leq$ .05. | | | | | | |

| Table 7  *Mini-K Bifactor ESEM Loadings (Bi-Geomin Rotation)* | | | | | | | |
| --- | --- | --- | --- | --- | --- | --- | --- |
|  | Insight, planning, and control | Mother/  father rel. quality | Pair-bonding | Family support | Friends support | Community involvement | K |
| Item 2 | 0.467* | -0.059 | -0.049 | -0.072 | 0.076 | -0.111* | 0.351* |
| Item 3 | 0.597* | 0.069 | 0.064 | 0.019 | 0.015 | 0.081* | 0.125* |
| Item 4 | 0.636* | 0.026 | -0.039 | 0.038 | -0.100* | 0.017 | 0.318* |
| Item 5 | 0.000 | -0.041 | -0.329* | -0.028 | -0.012 | -0.052 | 0.686* |
| Item 7 | 0.042 | 0.756* | 0.032 | 0.058 | 0.043 | -0.101* | 0.220* |
| Item 8 | -0.002 | 0.662* | -0.019 | -0.05 | 0.017 | 0.113* | 0.120* |
| Item 11 | -0.010 | -0.003 | 0.608* | -0.018 | 0.016 | -0.158* | 0.560* |
| Item 12 | -0.010 | -0.004 | 0.319 | -0.006 | -0.095* | 0.241* | 0.555* |
| Item 13 | -0.089* | 0.072 | 0.055 | 0.666* | -0.054 | 0.031 | 0.288* |
| Item 14 | -0.023 | 0.062* | -0.075* | 0.899* | -0.014 | -0.070* | 0.249* |
| Item 15 | 0.097* | -0.128* | 0.031 | 0.835* | 0.079* | 0.054 | 0.223* |
| Item 16 | -0.023 | 0.048 | -0.083 | -0.001 | 0.754* | 0.055 | 0.291* |
| Item 17 | -0.058* | 0.066* | -0.008 | 0.045 | 0.808* | 0.022 | 0.390* |
| Item 18 | 0.072* | -0.036 | 0.065 | 0.015 | 0.760* | -0.013 | 0.433* |
| Item 19 | 0.033 | -0.017 | 0.049 | 0.003 | 0.228* | 0.524* | 0.299* |
| Item 20 | 0.002 | 0.039 | -0.012 | 0.053 | -0.101* | 0.627* | 0.232* |
| *Note*: Table displays standardized loadings. **p* $\leq$ .05. Expected loadings are highlighted in dark gray. Cross-loadings $\geq$ .10 are highlighted in light gray. General factor loadings from target rotated solution in K (target) column. | | | | | | | |
